# Salt Plug Formation Caused by Decreased River Discharge in a Multi-channel Estuary

**DOI:** 10.1038/srep27176

**Published:** 2016-06-03

**Authors:** Dinesh Chandra Shaha, Yang-Ki Cho

**Affiliations:** 1 Department of Fisheries Management, Bangabandhu Sheikh Mujibur Rahman Agricultural University, Gazipur 1706, Bangladesh; 2 School of Earth and Environmental Science/Research Institute of Oceanography, Seoul National University, Seoul 151-742, Korea

## Abstract

Freshwater input to estuaries may be greatly altered by the river barrages required to meet human needs for drinking water and irrigation and prevent salt water intrusion. Prior studies have examined the salt plugs associated with evaporation and salt outwelling from tidal salt flats in single-channel estuaries. In this work, we discovered a new type of salt plug formation in the multi-channel Pasur River Estuary (PRE) caused by decreasing river discharges resulting from an upstream barrage. The formation of a salt plug in response to changes in river discharge was investigated using a conductivity-temperature-depth (CTD) recorder during spring and neap tides in the dry and wet seasons in 2014. An exportation of saline water from the Shibsa River Estuary (SRE) to the PRE through the Chunkhuri Channel occurred during the dry season, and a salt plug was created and persisted from December to June near Chalna in the PRE. A discharge-induced, relatively high water level in the PRE during the wet season exerted hydrostatic pressure towards the SRE from the PRE and thereby prevented the intrusion of salt water from the SRE to the PRE.

The natural variability of river inflows to estuaries has been greatly modified by dam construction for irrigation, drinking water and energy production. Changes in river discharge alter estuarine circulation, stratification, flushing time, and the transport of dissolved and particulate materials^1^,^2^,^3^. Therefore, understanding the response of estuarine circulation to the temporal changes in river discharge is very important.

River discharge in tropical regions differs substantially from those in temperate and wet tropical regions. Indeed, river discharge into tropical estuaries is often highly seasonal, with very large flows in the wet season being followed by five to ten months of negligible discharge in the dry season^4^. In the hot, dry season, evaporation exceeding precipitation results in a salinity maximum zone near the mouth of tropical estuaries that is referred to as a salt plug^5^. The salinity decreases seaward and landward from this salt plug, similar to inverse and positive estuaries, respectively^1^,^5^. This type of system is relatively uncommon among the world’s estuaries. It was first identified in the Alligator River of Australia in 1986, where evaporation is responsible for the formation of salt plugs^5^. A salt plug that was not formed by evaporation was identified at low tide in the Norman River of Australia^6^. Salt outwelling from the tropical tidal salt flats of the Norman River during the hot, dry months of summer (October-November) coinciding with negligible runoff was responsible for this salt plug. Salt plugs isolate the upper reaches of estuaries from the coastal waters for a few months of the year and inhibits, or even prevents, the flushing of estuarine waters with ocean waters^5^. Consequently, suspended and soluble substances may be trapped for long periods in the dry season. The ecological implications of such long-term trapping include the creation of anoxic or hypoxic conditions^7^. Minimal information exists in the literature on the formation of salt plugs, and studies have been limited to single channel estuaries^5^,^6^.

The Pasur River Estuary (PRE) is a complex, tropical multi-channel estuarine system connected by the Chunkhuri, Pankhali and Batiaghata Channels to the Shibsa River Estuary (SRE) upstream (Fig. 1b,c). The PRE and the SRE experience different tidal-phase lags upstream^8^, seasonal forcing from river discharge and precipitation regimes^8^,^9^. The travel time of the tide from the estuarine mouth (at Hiron Point) to Nalianala in the Shibsa River (Fig. 1b) is approximately 2 hours, whereas that to Mongla Port in the Pasur River from Hiron Point is approximately 2.5 hours^8^, even though the distances are approximately the same (~70 km). The PRE has a direct link with the Ganges, the principal freshwater source upstream, whereas the SRE does not. The Ganges, which is the third largest river by discharge in the world, was unregulated prior to the construction of the Farakka barrage in 1975. Subsequently, 1134 m^3^ sec^−1^ of water flow was diverted from the Ganges to the Bhagirathi-Hooghly River to aid navigation in the latter during the dry season^9^. This diversion decreased the average dry season flow in the Ganges from 3114 m^3^ sec^−1^ during the pre-Farakka period to 2010 m^3^ sec^−1^ during the post-Farakka period^9^,^10^. This diversion of freshwater from the Ganges at Farakka also reduced the dry-season discharge in the Gorai River, the major distributary of the Ganges and the main freshwater source of the PRE upstream, from a pre-Farakka mean flow of 190 m^3^ sec^−1^ in 1973^9^,^10^ to post-Farakka mean flows of 51 m^3^ sec^−1^ in 1977 and 10 m^3^ sec^−1^ in 2008^11^. Consequently, freshwater flow has declined in the SRE by almost 60% since 1977^8^ because the SRE is connected by lateral channels to the PRE. Since 1977, a sharper increase in tidal range has occurred at Nalianala on the Shibsa River than at Mongla in the Pasur River^8^. Thus, water levels in the Pasur River are highly dependent on the freshwater inflow from upstream, whereas those in the Shibsa River are highly dependent on the tide (Fig. 2a,b). As a result, the salinity in the SRE is notably higher than that in the PRE in the dry season (December to July, Fig. 2c).

Understanding the salinity variation in estuaries under different forcing events, such as tide, extreme drought, climate change and human interventions, is important for the management of estuarine water resources, the design of water-supply systems and the protection of diverse estuarine and mangrove species^4^,^12^,^13^,^14^,^15^. Therefore, salt water intrusion in the PRE has received substantial attention in recent years because of increases in the magnitude and frequency of salt water intrusion upstream that directly influenced the economic development of the southwest region of Bangladesh^9^,^16^. Most previous studies focused on analysing the relationships between discharge and salinity in the PRE and SRE^9^,^10^,^16^,^17^,^18^. The salt water intrusion has extended as far as ~164 km from the estuarine mouth (at Hiron Point) to a head at Lohagara, Narail, during the spring tide in the dry season (March 2014), with a salinity of 0.19^19^. Salt water intrusion leads to shortages of drinking and irrigation water^20^, decreased rice production^9^, inadequate industrial supply^10^ and adversely affects the habitats of freshwater fish^16^,^21^. A few studies of the surface-water quality^22^, nutrient distributions^23^ and hydrology of mangrove ecosystems^8^ have been conducted in the PRE. However, previous studies have typically omitted hydrographic details, such as longitudinal and time-series depth profiles of water temperature, salinity, and density, which are necessary for understanding the hydrodynamics and causes of salt water intrusion upstream through this complex multi-channel estuarine ecosystem.

Salinity distribution in estuaries is mainly determined by estuary shape, tide, river discharge, evaporation and salt outwelling from tidal salt flats^1^,^2^,^4^,^5^,^6^,^12^. Previous studies reported that salt plugs can be created near the mouths of tropical estuaries during the dry and hot season because of evaporation and salt outwelling from tidal salt flats^5^,^6^. However, the contribution of connecting channels to the exportation of saline water from one multi-channel estuary to another, with the subsequent creation of salt plugs during the dry season when river discharge is negligible, has received very little attention. Moreover, most low-inflow estuarine systems have not yet been described or the low-inflow periods have been ignored while describing wetter periods in a given estuary^24^. In this study, we investigated how a salt plug formed and disappeared in a multi-channel estuarine system in response to changes in river discharge due to an upstream barrage. High-resolution data measured with a conductivity-temperature-depth (CTD) profiler facilitated an understanding of the effects of discharge-induced relative water level variation between the PRE and the SRE. During the dry season, a salt plug is formed in the PRE because of the export of salt water from the SRE. By contrast, during the wet season, the salt plug disappears because of the prevention of salt water intrusion from the SRE to the PRE.

## Results

### Salt plug formation during the dry season

The spatial and temporal distributions of salinity are useful indicators for understanding the hydrodynamic parameters of estuaries, including stratification^25^,^26^, flushing^27^, the distribution patterns of ecological parameters^28^, and salt plugs^5^,^6^. The longitudinal vertical salinity sections of the PRE during the dry season show a salt plug near Chalna, 34 km upstream from Harbaria (Fig. 3a). The salinity profiles indicate a gradual reduction in the salinity of the salt plug landward (from Chalna to Rupsha Bridge), similar to a positive estuary, and a gradual salinity decrease seaward of the salt plug (from Chalna to Harbaria), similar to an inverse estuary. The maximum difference in the salinity between Harbaria and Chalna was as great as 3. The salinity and density gradients decreased seaward from this salt plug area (from Chalna to Harbaria) to 5.4 × 10^−2^ km^−1^ and 3.8 × 10^−2^ kg m^−3^ km^−1^, respectively, during the dry season. Landward of this salt plug area (from Chalna to Rupsha Bridge), the salinity gradient increased to 14.8 × 10^−2^ km^−1^ at the upstream limit of the longitudinal section, producing a steep horizontal density gradient of nearly 13.6 × 10^−2^ kg m^−3^ km^−1^. The salinity gradient is small in the downstream direction because of the tide and large in the upstream direction because of river discharge^2^. This pattern of longitudinal salinity distribution is consistent with density-driven flows in salt plug estuaries^4^,^5^,^29^.

The salt plug existed for several months in the PRE when small river discharge occurred during the dry season (<30 m^3^ s^−1^) (Fig. 3a) and essentially isolated the upper reaches of the estuary from the coastal water. By contrast, the waters of the downstream estuary exchanged freely with the Bay of Bengal. The December salinity section clearly indicates a transition period in reversing the PRE from a typical estuary (wherein salinity decreases in the upstream direction from the downstream area) during the wet season (Fig. 3b) to a salt plug estuary during the dry season (Fig. 3a), as the river discharge decreased. The salt plug area was small (approximately 10 km) in December. However, a salt plug of 30 to 40 km (between Joymoni and Batiaghata) was observed in the PRE in the other dry season months. The salt plug formed near Chalna in the PRE during the dry season because of the exportation of saline water from the SRE to the PRE through the Chunkhuri Channel may be due to the variation in the pressure gradient^1^,^29^ produced by the water level slope between the SRE and the PRE (Fig. 2b) and the density difference between the Chunkhuri Channel and the PRE (Supplementary Fig. S1a), as explained in detail in the Discussion section below. The variability of the salt plug area in the PRE during the dry season resulted from spring-neap tidal variation because the freshwater discharge was negligible upstream of the PRE during the dry season.

### Salt plug disappearance during the wet season

Hydrographic surveys performed along the PRE during the wet season showed a gradual decrease in salinity from Harbaria to Batiaghata (Fig. 3b). This salinity distribution resembles that of typical estuaries^1^,^2^,^26^,^30^. The maximum salinity value of 3 at Harbaria decreased to 0.13 at Batiaghata in July 2014. The horizontal salinity and density gradients were 2.5 × 10^−2^ km^−1^ and 2.2 × 10^−2^ kg m^−3^ km^−1^ between Harbaria and Chalna and 0.3 × 10^−2^ km^−1^ and 0.1 × 10^−2^ kg m^−3^ km^−1^ between Chalna and Batiaghata, respectively. The river discharge was 300 times greater upstream of the PRE during the wet season than during the dry season (Fig. 2a). As a result, discharge-induced strong gravitational flow during the wet season not only advected the salt plug from the PRE to the Bay of Bengal (Fig. 3a,b) but also prevented the intrusion of salt water from the SRE to the PRE through the Chunkhuri Channel (Supplementary Fig. S1b) due to the relatively higher water level associated with the river discharge in the PRE (Fig. 2a,b), as described in detail in the Discussion section below. Therefore, the circulation resembled that of a typical estuary during the wet season rather than the inverse estuarine circulation observed during the dry season^1^,^2^,^30^.

### Temporal variations in salinity during the wet and dry seasons

The time-depth distributions of salinity recorded at Mongla Port during the spring and neap tides of the wet season (July and August) are shown in Fig. 4a. The monthly mean river discharges were 755 m^3^ s^−1^ and 2337 m^3^ s^−1^ in July and August 2014, respectively (Fig. 2a). At high tide, the maximum salinities were 1.1 in July and 0.2 in August at Mongla Port. The difference in salinity between two subsequent wet season months was approximately 0.9 and appeared to be primarily due to the difference in freshwater input because the river discharge was 209% higher in August than in July. The diurnal variation in maximum salinity at high tide and minimum salinity at low tide was consistent with classical estuaries, wherein the highest salinity is observed at slack high tide because of the intrusion of saline water from the ocean caused by the tide^1^,^2^,^26^,^30^. This finding also indicates that the inflow of saline water from the Shibsa River to the Pasur River was hindered during the wet season by discharge-induced, relatively high water levels in the PRE (Fig. 2b). As a result, the PRE showed typical estuarine characteristics during the wet season.

The time-depth distributions of salinity during the spring and neap tides in April and May 2014 (the dry season) are shown in Fig. 4b. The monthly mean river discharges were 4 m^3^ s^−1^ in April and 10 m^3^ s^−1^ in May. The salinity difference between high and low tides at Mongla Port in the PRE was approximately 1.5 during the spring tide. A minimum salinity of less than 15 was observed in consecutive high tides, which occurred during the daytime and nighttime successively, whereas a maximum salinity exceeding 16 was found during consecutive low tides, which occurred during nighttime and daytime successively. This salinity distribution is contrary to that in classical estuaries, where the highest salinity is observed at slack high tide resulting from the intrusion of ocean water because of the tide^1^,^26^,^30^. At slack low tide, the salinity was maximized because of the presence of a salt plug further up the Mongla Port during the dry season. The increase in salinity during low water at Mongla Port is assumed to be entirely due to the retreat of salt water from upstream to Mongla Port. Thus, the salt plug shifted upstream or downstream from Mongla Port according to the tide during the dry season. The highest salinity at slack low water was also found during the neap tide in the dry season because of the presence of a salt plug upstream of Mongla Port (Fig. 4b). During the neap tide, the minimum salinity was <15 at high tide, and the maximum was >16 at low tide. This salinity distribution during the neap tide is also contrary to that in classical estuaries^1^,^26^,^30^.

## Discussion

In this study, we investigated the influence of the discharge-induced relative water level variation between the PRE and the SRE. During the dry season, a salt plug forms in the PRE because of the export of saline water from the SRE. By contrast, during the wet season, the salt plug disappears because of the prevention of salt water intrusion from the SRE.

The vertical salinity and density sections during the dry season show an export of saline water from the Chunkhuri Channel to the PRE (Fig. 5a and Supplementary Fig. S1a). The salinity section in December implies an initiation of salt water intrusion from the SRE to the PRE through the Chunkhuri Channel during the transition from the wet season to the dry season when the monthly median river discharge was 21 m^3^ s^−1^. An increase in salinity along the Chunkhuri Channel from Chalna was observed during the dry season. The horizontal gradients of salinity and water density during the dry season were 37.8 × 10^−2^ km^−1^ and 33.2 × 10^−2^ kg m^−3^ km^−1^ in the Chunkhuri Channel and 10.1 × 10^−2^ km^−1^ and 8.7 × 10^−2^ kg m^−3^ km^−1^ in the PRE, respectively. Moreover, the water level is relatively higher at Nalianala in the Shibsa River than at Mongla Port in the Pasur River (both locations are very close to the Chunkhuri Channel) during the dry season (Fig. 2b). The pressure gradient is affected by the density difference between riverine and oceanic waters and the water level slope that develops between the river and the ocean^29^. Therefore, the gravitational flow produced by the density difference between the Chunkhuri Channel and the PRE (Fig. 5a and Supplementary Fig. S1a) and the water level slope between the SRE and the PRE (Fig. 2b) advances towards the PRE from the SRE during the dry season. As a result, the density gradient and the water level variation between the SRE and the PRE act in combination to facilitate the unidirectional export of salt water throughout the water column from the SRE to the PRE via the Chunkhuri Channel during the dry season (Fig. 5a and Supplementary Fig. S1a). In the peak dry season (June), density-induced gravitational flow becomes stronger in the Chunkhuri Channel (44.3 × 10^−2^ kg m^−3^ km^−1^) than in the PRE (9.1 × 10^−2^ kg m^−3^ km^−1^). Therefore, water with salinity >21.8 flows from the Chunkhuri Channel toward the PRE (Fig. 5a). Consequently, the salt plug can form near Chalna in the PRE over a 30-km to 40-km area (from Joymoni to Batiaghata) during the dry season (Fig. 3a). The tide moved the relative position of this salt plug back and forth in the PRE during the dry season (Fig. 4b). This salt plug’s development process differed from that of salt plugs produced by evaporation^5^,^31^.

By contrast, the vertical salinity and density sections during the wet season in the Chunkhuri Channel (Fig. 5b and Supplementary Fig. S1b) show a typical two-layer circulation produced by density gradients and the water level slope associated with the river discharge^1^,^29^. The salinity difference between the SRE and the PRE is >8 during the transition from the dry season to the wet season (July) (Fig. 5b). Wet season observations also show higher salinity and density gradients (32.9 × 10^−2^ km^−1^ and 24.3 × 10^−2^ kg m^−3^ km^−1^, respectively) in the Chunkhuri Channel relative to those in the PRE (1.4 × 10^−2^ km^−1^ and 1.1 × 10^−2^ kg m^−3^ km^−1^, respectively). The vertical profiles of salinity and density clearly indicate that the longitudinal density gradient drives a net volume near-bottom inflow to the PRE from the SRE and a stronger surface outflow to the SRE from the PRE than the near-bottom inflow that occurs in response to the supplementary freshwater in the PRE. This circulation is induced by the volume of fresh water added to the PRE from upstream during the wet season. Riverine waters, which are less dense than oceanic waters, are forced to flow seaward^29^. Because the water in the Chunkhuri Channel flowing from the SRE is denser than the water at Chalna in the PRE, the water level at the limit of the salt water intrusion in the Chunkhuri Channel at Chalna in the PRE is set up slightly above the mean water level during the wet season (Fig. 2b). This variation is because the river discharge is ~300 times greater than in the dry season. The resultant hydrostatic pressure near the water surface at Chalna in the PRE is directed towards the SRE. Thus, strong counteraction between density-driven and discharge-induced gravitational flow occurred in the Chunkhuri Channel at the beginning of the wet season (July), and the higher water level associated with river discharge in the PRE prevented the intrusion of saline water from the Chunkhuri Channel to the PRE during the wet season (Fig. 5b).

In addition, the discharge-induced gravitational flow during the peak wet season (August and October) became dominant over the density-driven flow (the density gradient decreased to 0.7 × 10^−2^ kg m^−3^ km^−1^ in August and 7.3 × 10^−2^ kg m^−3^ km^−1^ in October from 72.6 × 10^−2^ kg m^−3^ km^−1^ in July) in the Chunkhuri Channel that prevented saline water intrusion from the SRE to the Chunkhuri Channel (Fig. 5b). As a result, no salt plug could develop in the PRE during the entire wet season. Moreover, the salt plug that formed in the PRE during the dry season was advected to the Bay of Bengal from the PRE during the wet season due to the strong discharge-induced gravitational flow in the upstream area (Fig. 3b). Therefore, the circulation in the PRE resembled that of a typical estuary during the wet season.

A conceptual flow diagram describing the dry and wet seasons is shown in Fig. 6. During the dry season, the water level is higher at Nalianala in the SRE than at Mongla Port in the PRE (Fig. 2a,b). This relative water level variation can exert hydrostatic pressure towards the PRE from the SRE. As a result, saline water can flow from the SRE to the PRE through the Chunkhuri Channel and create a salt plug near Chalna during the dry season (Fig. 3a). The tide then moves the salt plug upstream in the PRE because the tidal phase lag is half an hour later in the PRE than in the SRE. A substantial portion of this salt plug retreated to the Mongla Port during the subsequent ebb tide (Fig. 4b) because the PRE is wider than the Chunkhuri Channel. Therefore, this salt plug shifted up and down the estuary because of tidal action during the dry season (Fig. 4b). By contrast, the discharge-induced higher water level in the PRE exerted hydrostatic pressure from the PRE towards the SRE during the wet season and prevented the intrusion of saline water from the SRE to the PRE (Fig. 5b). Additionally, the salt plug of the PRE was flushed out to the Bay of Bengal during the wet season due to strong discharge-induced gravitational flow in the upstream area (Fig. 3b).

The longitudinal and time series salinity and density profiles of the PRE and the Chunkhuri Channel suggest a persistence of the salt plug in the PRE during the dry season. It is likely that the system behaves similar to a salt plug estuary^5^,^6^. One noteworthy feature of this salt plug estuary is the separation of the circulation driven by the river input of freshwater from the circulation involving exchange with the coastal water. The exchange of water between the two sides of the salt plug occurs via turbulent diffusion as the mean flow approaches zero at the salt plug^31^. Therefore, almost no water passes through the salt plug, and very little river water even reaches the sea during the dry season^5^,^29^.

The port authority frequently conducts dredging activities to facilitate navigation near Mongla Port because of the relatively high sedimentation in the port area^19^. This high sedimentation rate may be attributable to the presence of the salt plug near Mongla Port because the flows may be near-zero in the salt plug area^31^, which could induce sedimentation. System flushing of the PRE during the dry season was also weaker than flushing during the wet season^32^. Low river discharge and salt plug conditions can lower the flushing rate during the dry season in the PRE. Therefore, the salt plug may trap nutrients, sediments, and other organic and inorganic substances in the PRE for longer periods during the dry season, potentially creating water quality problems (Supplementary Fig. S2). The low dissolved oxygen level (dropped <5.0 mg L^−1^) is a sign of possible pollution in the salt plug area of the PRE in the dry season, indicating that nearby aquatic life is subjected to stress. A recent study on fish biodiversity in the PRE identified 14 threatened species out of 95 finfish species^33^. Therefore, the salt plug may have important consequences for the management of this estuary because all materials, including mangrove detritus introduced into the estuary on the river side, may accumulate in the salt plug area during the dry season and result in low concentrations of dissolved oxygen.

## Methods

**Figure 1:**
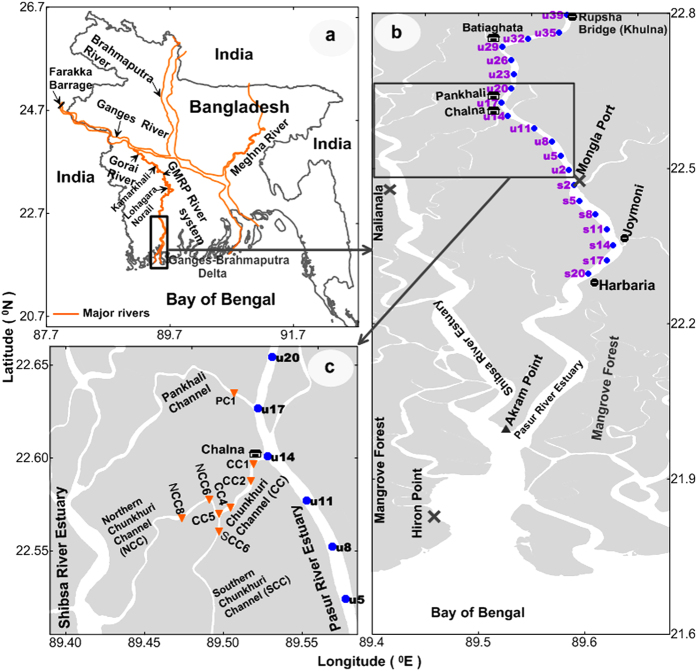
Study site locations. (**a**,**b**) Map of the complex topographical features of the multi-channel Pasur River-Shibsa River estuarine system in the southwestern coastal zone of Bangladesh. (**b**) Conductivity-Temperature-Depth (CTD) recorder stations are shown as blue solid circles (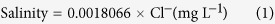) in the Pasur River. The Xs (**X**) denote the locations of the tidal stations at Hiron Point, Mongla Port and Nalianala. An X also indicates the time-series CTD station at Mongla Port. (**c**) The Chunkhuri and Pankhali Channels connect the Pasur River to the Shibsa River approximately 70 km upstream from Hiron Point. The Chunkhuri Channel bifurcates into two branches (north and south) at approximately 5 km (CC5) from Chalna. CTD stations are shown as orange solid triangles (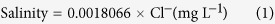 ) in the Chunkhuri and Pankhali Channels. The map was generated using Golden Software Surfer 9.0 (www.goldensoftware.com).

The Pasur River is the longest and most important river for the world’s largest Sundarbans mangrove ecosystem^34^, which is located in the southwestern part of the Ganges-Brahmaputra delta of Bangladesh. The Pasur River splits into two branches, the Shibsa River and the Pasur River, at Akram Point after entering from the Bay of Bengal (Fig. 1b). The Shibsa River is deeper than the Pasur River^8^. The Chunkhuri Channel connects the Pasur River to the Shibsa River approximately 70 km upstream from Hiron Point, at Chalna (Fig. 1c). The interconnecting channel complicates the morphology of the Pasur-Shibsa estuarine system (Fig. 1b) and likely shapes the complex water circulation. The hydrological regimes of the multi-channel PRE-SRE system largely depend on the upstream discharge of the Gorai River, local rainfall, and the tide in the Bay of Bengal. The PRE is directly linked to the main freshwater source of the Ganges through the Gorai-Madhumati-Rupsha-Pasur (GMRP) river system to the Bay of Bengal, whereas the SRE is connected by lateral channels to the PRE at Chalna, Pankhali and Batiaghata (Fig. 1b). As a result, the SRE experiences high salinization relative to the PRE in the dry season because of the lack of freshwater runoff and rainfall^8^. The Ganges originates in the Himalayas and flows through India and Bangladesh and empties into the Bay of Bengal through the GMRP river system (Fig. 1a).

**Figure 2:**
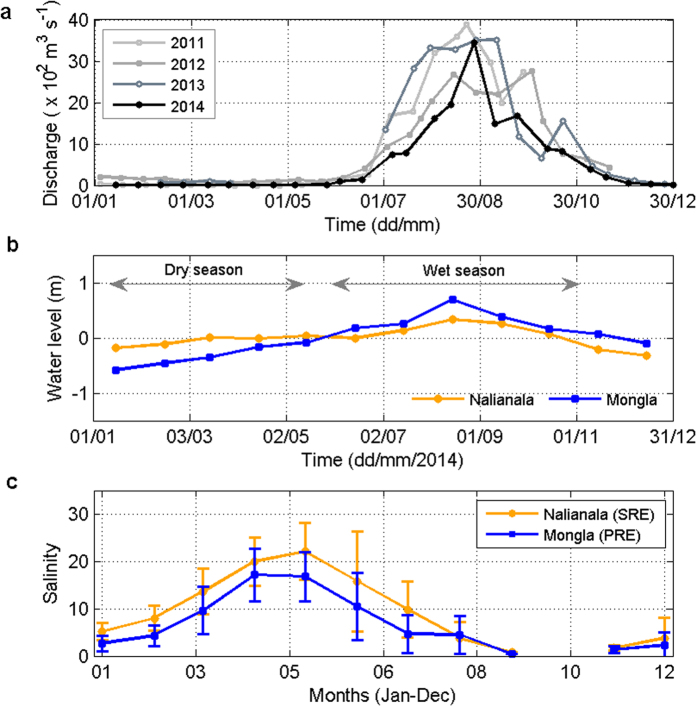
River discharge, water level and salinity. (**a**) Annual variation in river discharge measured fortnightly at Kamarkhali station upstream of the Pasur River Estuary (PRE) from 2011 to 2014. (**b**) Comparative monthly mean water level variation between Nalianala in the Shibsa River Estuary (SRE) and Mongla Port in the PRE after removing the mean sea level at each station. The water level in the Pasur River is highly dependent on the freshwater inflow from upstream, whereas that is the Shibsa River is less dependent on upstream flow because of the lack of a direct connection with the main freshwater source. (**c**) Comparison of monthly mean salinity at Nalianala (SRE) and Mongla (PRE) from November 2000 to June 2013. The salinity in the SRE was significantly higher than that in the PRE in the dry season (December to July).

**Figure 3:**
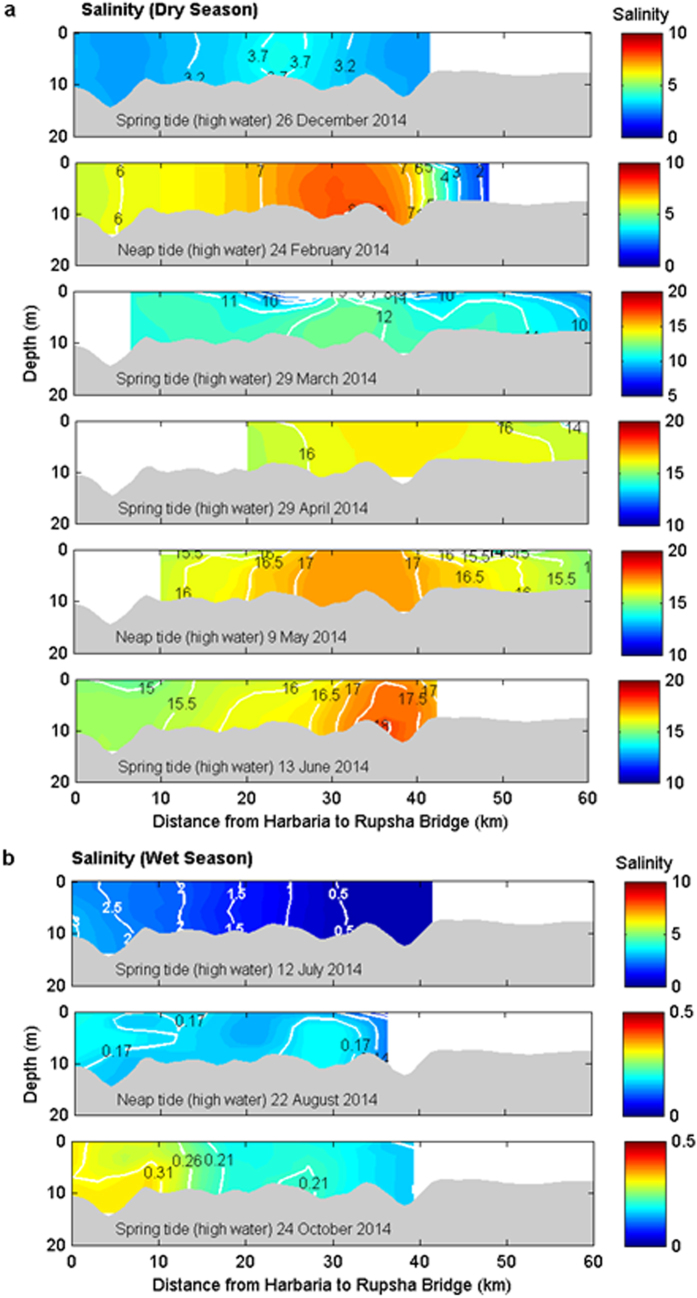
Longitudinal salinity sections in the Pasur River Estuary. (**a**) The vertical salinity sections obtained along the main axis of the Pasur River Estuary during the dry season (December, February, March, April, May and June). A salt plug developed near Chalna (34 km upstream of Harbaria). This salt plug started to develop in transit during the dry winter season (December and February). (**b**) Same as Fig. 3a but for the wet season (July, August, and October). The salt plug disappeared, and a typical estuarine system developed. Refer to Fig. 1b for the location.

**Figure 4:**
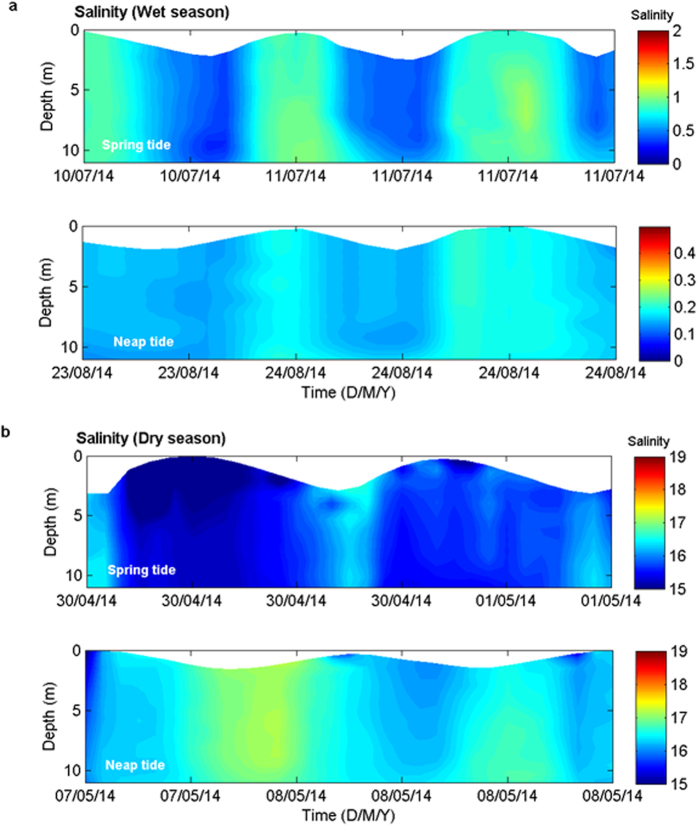
Time-depth distributions of the salinity profile. (**a**) Time-depth salinity distribution observed at Mongla Port during the spring and neap tides during the wet season (July and August 2014). The maximum salinity at high waters corresponds to that of classical estuaries during the wet season. (**b**) Same as Fig. 4a but for the dry season (April and May 2014). The maximum salinity at low waters is contrary to that of classical estuaries, where the highest salinity is observed at slack high tide. The highest salinity at slack low tide is assumed to be attributable to the retreat of salt water from the upstream part.

There are three distinct seasons^35^ in Bangladesh: a hot, humid, dry summer from March to June; a rainy monsoon season from July to October; and a dry winter from November to February. Normally, approximately 80 to 90% of the annual rainfall occurs in the monsoon or wet months. River discharge data from January 2011 to December 2014 were collected from a non-tidal discharge station (at 23.5396 °N and 89.5159 °E, Kamarkhali, Faridpur) on the Gorai River, which is operated by the Bangladesh Water Development Board (BWDB). River discharges are strongly seasonal. Most discharges occur during the wet season, which extends from July to October (Fig. 2a). By contrast, river discharge is negligible in the dry season (November to June). In addition, chloride (mg L^−1^) data for Nalianala (SRE) and Mongla (PRE) during high water from November 2000 to June 2013 were obtained from the BWDB to investigate the long-term variation in salinity (in terms of chloride) between the SRE and PRE in the dry and wet seasons, as shown in Fig. 2c. Because most anions in seawater or brackish water are chloride ions, salinity is determined based on the chloride concentration. The following formula is used^36^:


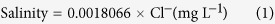


The tidal water level data for Mongla Port and Hiron Point were obtained from the Mongla Port Authority (Fig. 1b), and the water levels for Nalianala for 2014 were provided by the BWDB. Tides in the Bay of Bengal are predominantly semi-diurnal. The tidal range varied from 2.2 to 4.0 m at Mongla Port and from 1.6 to 3.0 m at Hiron Point during the neap and spring tides, respectively^37^. The tidal range is higher at Mongla Port than at Hiron Point. According to the classification of estuaries based on tidal range^2^,^30^, the PRE is a meso-tidal estuary.

The longitudinal and time series depth profiles of water temperature, salinity, and density were collected using a CTD profiler (Model: *In-situ* Aqua TROLL 200, *In-situ* Inc., Fort Collins, Colorado, USA), used for the first time in Bangladesh during this study, to examine the formation and disappearance of salt plugs under low and high river discharge conditions in the PRE. The longitudinal transects were taken along the main axis of the Pasur River from Harbaria to Rupsha Bridge over 60 km of river. No research vessel was available to collect data from the downstream area of this strongly tidally influenced estuary, especially southward from Harbaria to the estuary mouth, where speed boats or mechanized boats are not allowed to operate because of safety concerns. Nine longitudinal transects were taken at high water during both spring and neap tides, covering the dry and wet seasons from February to December 2014. A global positioning system (GPS) was used to obtain the exact locations along the estuary. The nominal distance between stations was approximately 3 km because of the low salinity gradient along the estuary. The time series depth profiles of salinity, temperature, and water density were collected with a CTD profiler from the Malancha vessel of the Mongla Port Authority at Jetty 5 of Mongla Port (Fig. 1b) during the spring and neap tides in the dry season (April and May 2014) and the wet season (July and August 2014) to investigate the hydrodynamic regime associated with the marked seasonal variability in river discharge.

**Figure 5:**
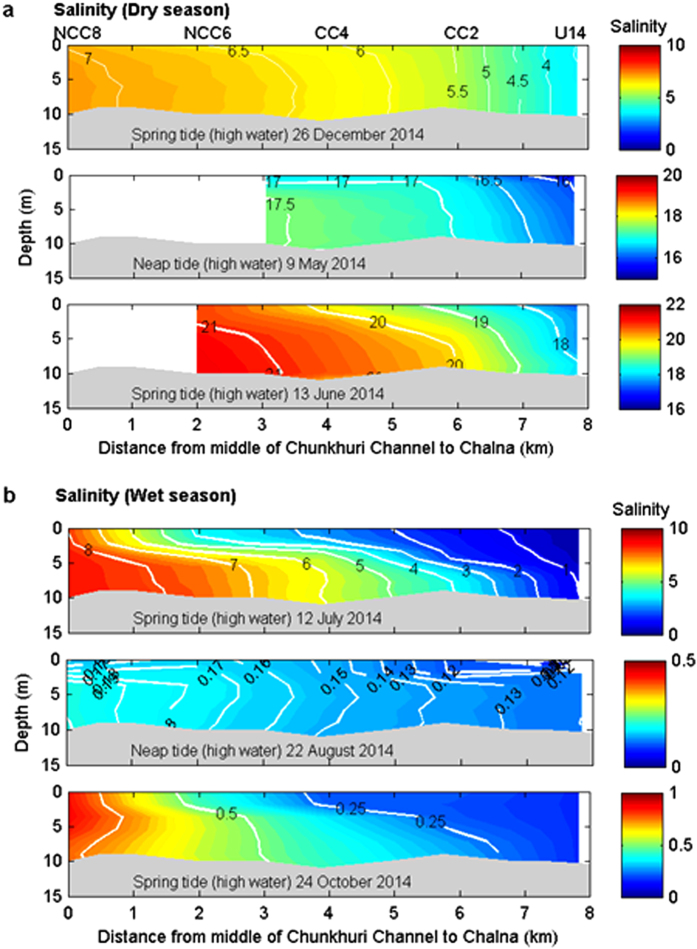
Longitudinal salinity section in the Chunkhuri Channel. The vertical salinity sections of the Chunkhuri Channel during the (**a**) dry and (**b**) wet seasons. (**a**) A *tongue-like intrusion* of saline water exists from Chunkhuri Channel to the Pasur River Estuary (PRE) during the dry season. (**b**) A strong counteraction between the *tongue-like intrusion* of saline water and the freshwater buoyancy force was observed during the transition from the dry season to the wet season (July) and prevents the intrusion of salt water from the Chunkhuri Channel to the PRE. Refer to Fig. 1c for the location.

**Figure 6:**
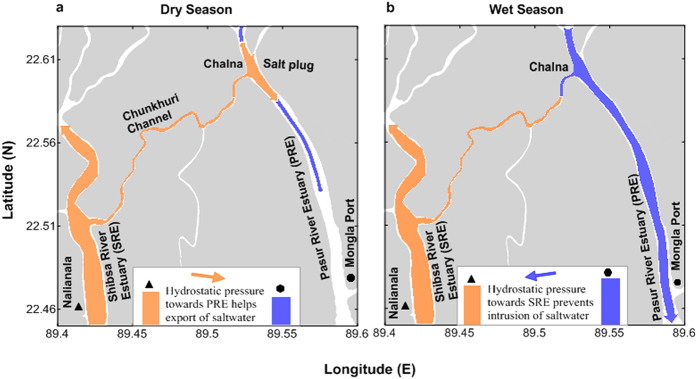
Conceptual diagram of salt plug formation and disappearance. (**a**) The relative water level variation between the SRE and the PRE during the dry season may exert hydrostatic pressure towards the PRE from the SRE and facilitate the export of salt water from the SRE to the PRE through the Chunkhuri Channel, creating a salt plug. This salt plug persists for several months (December-June). (**b**) The discharge-induced, relatively high water level in the PRE during the wet season exerts hydrostatic pressure towards the SRE from the PRE, thereby preventing the intrusion of salt water from the SRE to the PRE. In addition, the salt plug was flushed from the PRE to the Bay of Bengal during the wet season, and the PRE exhibits characteristics of a typical estuary. The map was generated using Golden Software Surfer 9.0 (www.goldensoftware.com).

The salt plug was first identified near Chalna in the PRE during the field campaign on 24 February, 2014. The longitudinal transect was repeated in March 2014 to investigate the existence of the salt plug near Chalna and advanced 66 km upstream from Chalna to observe the salinity distribution up to the limit of salt water intrusion (Supplementary Fig. S3). In the next cruise (April 2014), after confirming the existence of the salt plug near Chalna by spot checking, we advanced along both the Chunkhuri Channel and Pankhali Channel to approximately 1 km from the confluence of these channels to examine the source of high salinity water mass in the PRE. The bottom salinity (16.6) in the Pankhali Channel was similar to the confluence salinity, whereas the bottom salinity (16.8) in the Chunkhuri Channel was greater than the confluence salinity. Thereafter, we planned a longitudinal section in the Chunkhuri Channel from Chalna in the following cruises to investigate the occurrence of a salt plug nearby Chalna. In the next cruise (May 2014), the longitudinal transect was extended to approximately 5 km in the Chunkhuri Channel from Chalna, and a salinity of >17.5 was measured (Fig. 5a). However, the Chunkhuri Channel bifurcates into two branches (north and south) approximately 5 km from Chalna (Fig. 1c). Therefore, we advanced along both the southern and northern branches of the Chunkhuri Channel to approximately 1 km from the bifurcation point (CC5, Fig. 1c) in the next cruise (June 2014). The bottom salinity in the southern branch (SCC6, Fig. 1c) was 20.8, similar to the bottom salinity of the bifurcation point, whereas a bottom salinity of 21.43 was measured in the northern branch (NCC6, Fig. 1c) of the Chunkhuri Channel. It should be noted that the northern Chunkhuri Channel is deeper than both the southern Chunkhuri Channel and the Pankhali Channel because it is used for navigation between the SRE and the PRE. Thus, the northern Chunkhuri Channel was determined to be the water mass source with greater salinity. Therefore, the longitudinal CTD transect was taken up to approximately 8 km from Chalna in the northern Chunkhuri Channel and up to about 41 km upstream from Harbaria in the PRE, considering the high water slack period from July to December 2014, to investigate the causes underlying the formation and disappearance of the salt plug in the PRE.

## Additional Information

**How to cite this article**: Shaha, D. C. and Cho, Y.-K. Salt Plug Formation Caused by Decreased River Discharge in a Multi-channel Estuary. *Sci. Rep.* **6**, 27176; doi: 10.1038/srep27176 (2016).

## Supplementary Material

Supplementary Information
