# Supplementary material for: Salt Plug Formation Caused by Decreased River Discharge in a Multi-channel Estuary
Source: Sci Rep. 2016 Jun 3;6:27176. doi: 10.1038/srep27176 (PMC4891659; doi:10.1038/srep27176)
Supplement: Supplementary Information [file srep27176-s1.pdf]

SUPPLEMENTARY INFORMATION

Supplementary Figure S1-S3

Salt Plug Formation Caused by Decreased River Discharge in a Multi-channel Estuary

Dinesh Chandra Shaha[*,1,2], Yang-Ki Cho[2]

[1]Department of Fisheries Management, Bangabandhu Sheikh Mujibur Rahman Agricultural University, Gazipur 1706, Bangladesh

[2]School of Earth and Environmental Science/Research Institute of Oceanography, Seoul National University, Seoul 151-742, Korea

Corresponding author[*]

Phone: (02) 9205310-14 2494
Fax: (02) 9205333
Email: shaha.dinesh@gmail.com

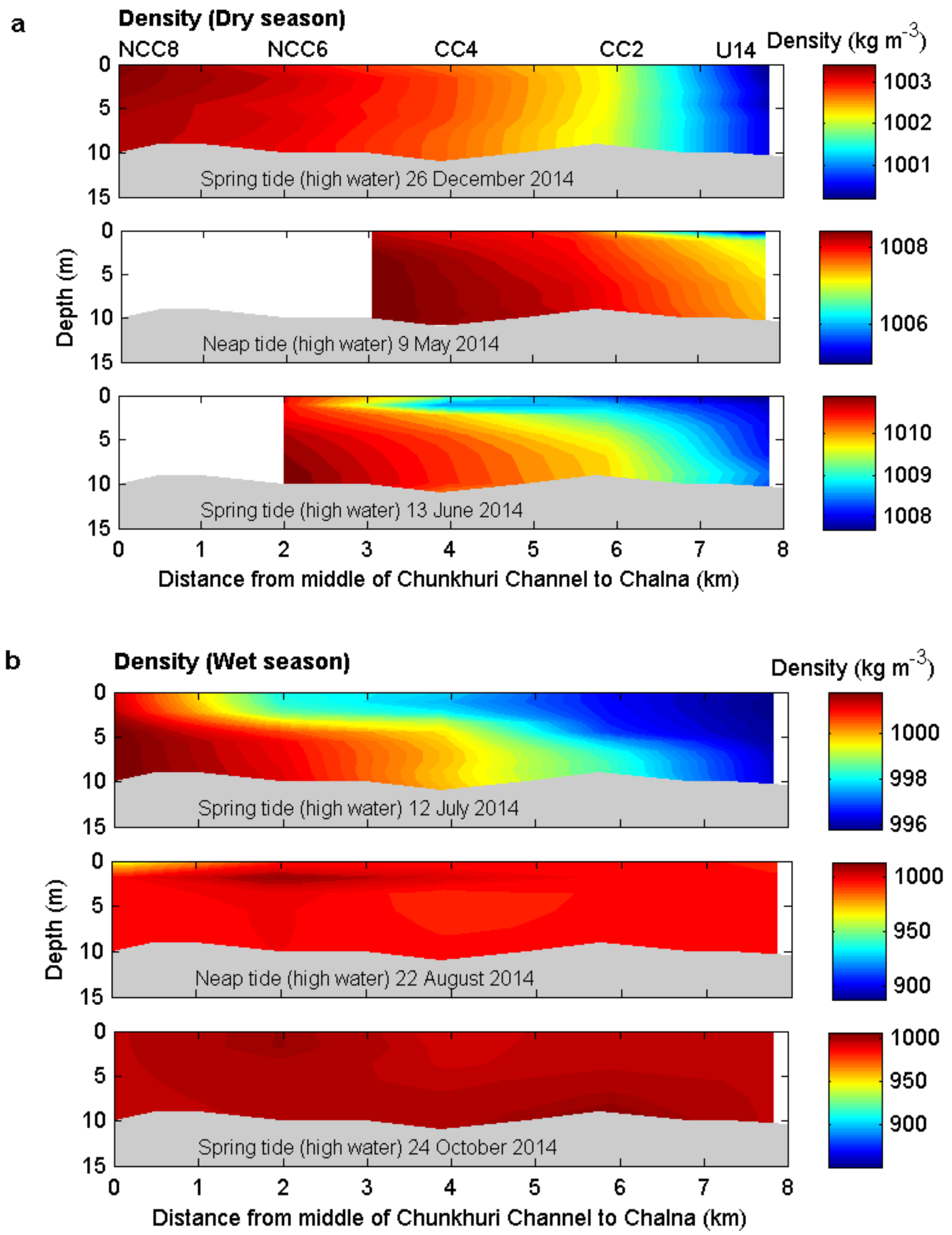

Supplementary Figure S1. Longitudinal density section in the Chunkhuri Channel. The vertical density sections of the Chunkhuri Channel during the (**a**) dry and (**b**) wet seasons. (**a**) A tongue-like intrusion of highly dense saline water exists from Chunkhuri Channel to the Pasur River Estuary (PRE) during the dry season. (**b**) A strong counteraction between the tongue-like intrusion of highly dense saline water and the freshwater buoyancy force was observed during the transition from the dry season to the wet season (July) and prevents the intrusion of highly dense salt water from the Chunkhuri Channel to the PRE. Refer to Figure 1c for the location.

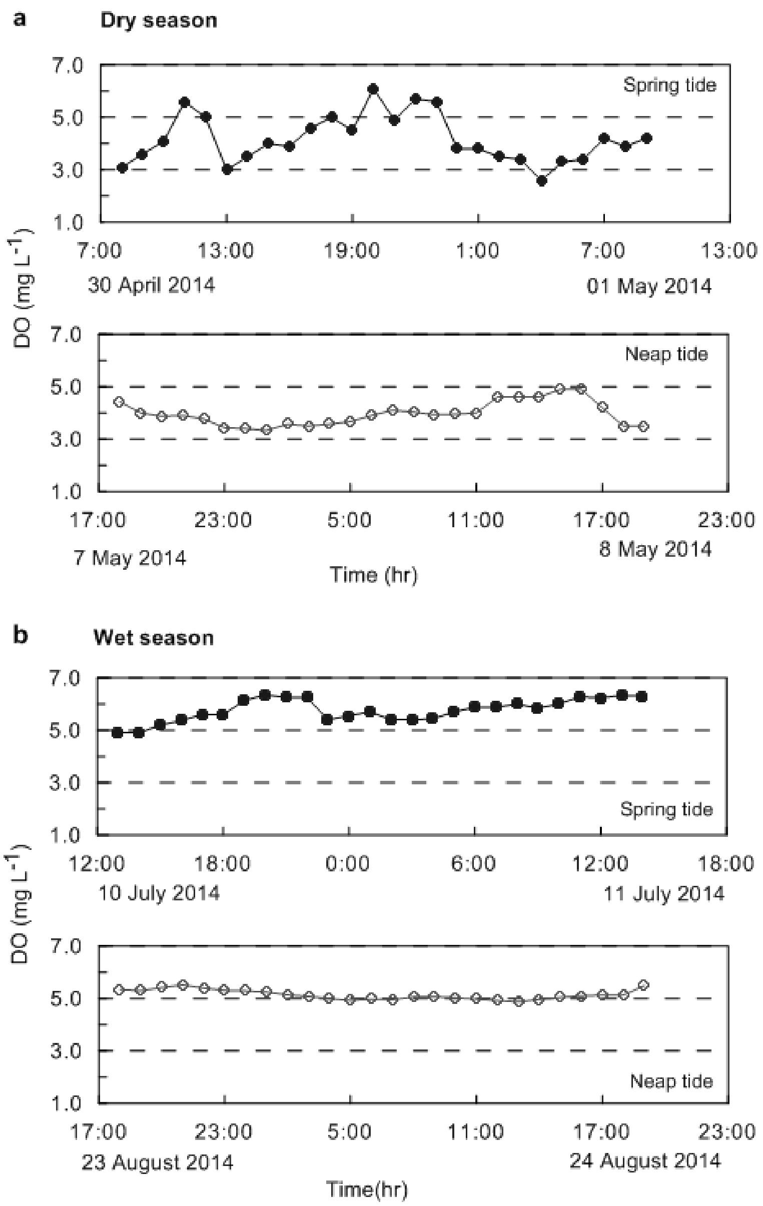

Supplementary Figure S2. Dissolved oxygen (mg L^{-1}). Diurnal variation of dissolved oxygen (DO) at surface water observed at Mongla Port during spring and neap tides in the **(a)** dry (April, May 2014) and (**b**) wet (July, August 2014) seasons. Dissolved oxygen is a basic requirement for a healthy aquatic ecosystem. **(a)** As DO levels in water of the salt plug area drop <5.0 mg L^{-1} during both spring and neap tides in the dry season, aquatic life is subjected to stress. Low DO is a sign of possible pollution in the salt plug area of the PRE. Refer to Figure 1b for the sampling location at Mongla Port.

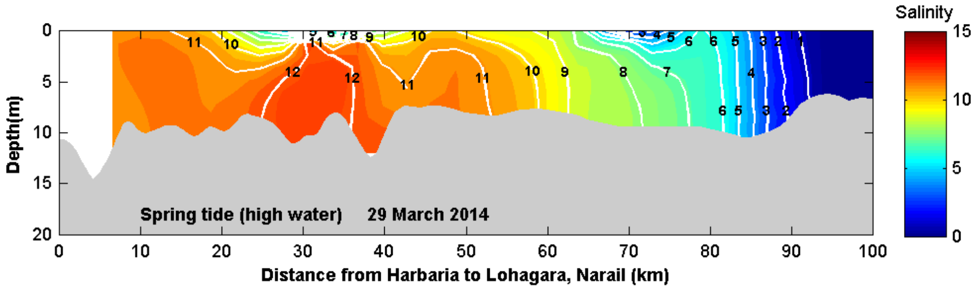

Supplementary Figure S3. Longitudinal salinity section in the Pasur River Estuary. The vertical salinity section obtained along the main axis of the Pasur River Estuary during the dry season (March 2014). A salt plug developed around Chalna, 34 km upstream from Harbaria.
